# Arthroscopic Determination of Cartilage Proteoglycan Content and Collagen Network Structure with Near-Infrared Spectroscopy

**DOI:** 10.1007/s10439-019-02280-7

**Published:** 2019-05-06

**Authors:** Jaakko K. Sarin, Olli Nykänen, Virpi Tiitu, Irina A. D. Mancini, Harold Brommer, Jetze Visser, Jos Malda, P. René van Weeren, Isaac O. Afara, Juha Töyräs

**Affiliations:** 1 Department of Applied Physics, University of Eastern Finland, Kuopio, Finland; 2 Diagnostic Imaging Center, Kuopio University Hospital, Kuopio, Finland; 3 Institute of Biomedicine, Anatomy, University of Eastern Finland, Kuopio, Finland; 4 Department of Equine Sciences, Faculty of Veterinary Medicine, Utrecht University, Utrecht, The Netherlands; 5 Regenerative Medicine Utrecht, Utrecht University, Utrecht, The Netherlands; 6 Department of Orthopaedics, University Medical Center Utrecht, Utrecht University, Utrecht, The Netherlands; 7 School of Information Technology and Electrical Engineering, The University of Queensland, Brisbane, Australia

**Keywords:** Equine, Deep learning, Neural networks, Arthroscopy, Mosaicplasty, Osteoarthritis, Post-traumatic osteoarthritis

## Abstract

Conventional arthroscopic evaluation of articular cartilage is subjective and insufficient for assessing early compositional and structural changes during the progression of post-traumatic osteoarthritis. Therefore, in this study, arthroscopic near-infrared (NIR) spectroscopy is introduced, for the first time, for *in vivo* evaluation of articular cartilage thickness, proteoglycan (PG) content, and collagen orientation angle. NIR spectra were acquired *in vivo* and *in vitro* from equine cartilage adjacent to experimental cartilage repair sites. As reference, digital densitometry and polarized light microscopy were used to evaluate superficial and full-thickness PG content and collagen orientation angle. To relate NIR spectra and cartilage properties, ensemble neural networks, each with two different architectures, were trained and evaluated by using Spearman’s correlation analysis (*ρ*). The ensemble networks enabled accurate predictions for full-thickness reference properties (PG content: *ρ*_*in vitro, Val*_= 0.691, *ρ*_*in vivo*_= 0.676; collagen orientation angle: *ρ*_*in vitro, Val*_= 0.626, *ρ*_*in vivo*_= 0.574) from NIR spectral data. In addition, the networks enabled reliable prediction of PG content in superficial (25%) cartilage (*ρ*_*in vitro, Val*_= 0.650, *ρ*_*in vivo*_= 0.613) and cartilage thickness (*ρ*_*in vitro, Val*_= 0.797, *ρ*_*in vivo*_= 0.596). To conclude, NIR spectroscopy could enhance the detection of initial cartilage degeneration and thus enable demarcation of the boundary between healthy and compromised cartilage tissue during arthroscopic surgery.

## Introduction

Osteoarthritis (OA) is a prevalent disease characterised by joint pain, restricted mobility, and instability of joint.^9^ Idiopathic OA is especially common among the elderly population and may be characterized by erosion of articular cartilage—the connective tissue that enables smooth and frictionless motion of the joints. As articular cartilage is aneural and avascular tissue,^29^ initial symptoms (e.g., joint pain) may occur in the later stages of the disease, making cartilage repair challenging. Development of OA can also be triggered by traumatic joint injuries.^8^ In contrast to idiopathic OA, post-traumatic OA (PTOA) can affect people of all ages.^35^ Local traumatic cartilage lesions will cause the surrounding tissue to experience higher stresses and strains,^38^ thus making the tissue more vulnerable to degeneration. Detection and intervention at the early disease stages (superficial proteoglycan (PG) loss and collagen damage) could be valuable for halting disease progression. Current methods to treat small chondral defects include arthroscopic debridement and lavage, osteochondral grafting, and autologous chondrocyte implantation.^37^ Although tissue engineering has advanced rapidly in the last decade, no engineered cartilage currently exists that matches the properties of native cartilage.^37^ In the event of insufficient repair, cartilage surrounding injury sites slowly degrades and will often exhibit signs of early to mid-stage degeneration (similar to those associated with early OA).^15^

Conventional imaging methods, such as radiography and magnetic resonance imaging, are well-suited for diagnosis of late stages of OA.^39^ These methods are, however, insufficient for detecting the early signs of cartilage damage. Ligamental tears and other traumatic injuries often require arthroscopic repair surgery. Currently, arthroscopic evaluation relies on visual assessment and scoring of cartilage lesions using established systems (e.g., International Cartilage Repair Society (ICRS)^7^) and manual probing with an arthroscopic hook. These methods are highly subjective^6^; thus, more quantitative techniques could be of great clinical significance.

Several quantitative techniques, including near-infrared spectroscopy (NIRS, 0.7–2.5 *µ*m) and mid-infrared spectroscopy (MIRS, 2.5–15 *µ*m), have been suggested for evaluating cartilage properties, such as biomechanical properties,^25^,^27^ cartilage thickness,^25^ and biochemical composition,^2^ in several *in vitro* studies. Clinical application of NIRS has only been presented in few studies^31^,^32^; however, these studies are limited by the use of simplistic univariate analysis techniques, in which the ratio of two spectral peaks at 1175 and 1425 nm is evaluated.^31^,^32^ These spectroscopic techniques enable non-destructive *in situ* evaluation without the need for sample extraction and are sensitive to specific bond vibrations common in biological materials.^10^ In conventional diffuse reflectance spectroscopy, samples are irradiated with light and the reflected and scattered light is collected and analysed. Changes in tissue composition contribute to the magnitude of light absorption, whereas tissue structure contributes to the magnitude of reflection and scattering. Although the MIR spectral region is more specific with relatively stronger absorption bands,^10^ the technique suffers from poor penetration depth into biological tissues.^20^ On the other hand, the NIR spectral region suffers from overlapping spectral features,^10^ making conventional univariate analysis ineffective. Currently, the availability of computational power and adaptation of state-of-the-art algorithms, such as neural networks (NNs), have enabled modelling of the relationship between NIR spectra and reference properties.^27^

Neural networks (NNs) are analytical techniques that mimic the function of the brain and consist of several layers of neurons. The input layer receives the data and passes it on to hidden layers, whereas the output layer defines network output. In the neurons of the hidden layers, the inputs are weighted and processed through different activations (e.g., rectified linear units (ReLu^18^)). With regression problems, linear activation is often used in the output layer. The aim of the network is to optimize the hyper-parameters (weights and biases) to minimize network error. Even shallow NNs have shown superiority in modelling the relationships between spectra and reference properties over principal component analysis (PCA) and partial least squares regression (PLSR)—the commonly applied analytical approaches in chemometrics.^12^,^19^ Deep learning applications, such as image recognition and classification, conventionally rely on convolutional NN (CNN) to process image data. For successful adaptation of deep learning, large training data are required for the modelling as limited data can often lead to overfitting. Several techniques have been introduced to minimize overfitting, including cross-validation,^36^ dropout layers,^34^ and early stopping.^41^ In cross-validation, an independent subgroup of data is withheld from the training data to monitor its prediction performance and thus to ensure a well-generalizable model. A dropout layer randomly deactivates neurons during training to make each neuron to learn broader features, whereas early stopping halts the training if no improvement is achieved after a certain number of iterations. Additional techniques for enhancing NN performance are data augmentation,^22^ in which the amount of training data is artificially increased (e.g., by the creation of slightly altered copies of the original data), and reduction of learning rate, in which learning rate is reduced to ensure finding the optimal solution.

We hypothesize that arthroscopic NIRS, combined with deep learning, can reliably predict cartilage PG content and collagen orientation angle in full-thickness and superficial regions of cartilage during *in vivo* arthroscopy. To test the hypothesis, *in vivo* and *in vitro* NIR spectra were acquired from tissue surrounding experimental cartilage repair sites in equine joints and compared to those acquired from matching anatomical locations in healthy equine joints. The surrounding tissue was investigated as the repair attempts were deemed unsuccessful and also to improve the currently challenging diagnosis of early cartilage degeneration. As reference, cartilage PG content and collagen orientation angle were determined with digital densitometry (DD) and polarized light microscopy (PLM), respectively. In addition, we applied one-dimensional CNNs (1D-CNNs) to investigate the relationship between cartilage NIR spectra and reference properties. Relative to earlier cartilage NIRS studies,^14^,^26^,^32^ the present study utilises a wider spectral region, adopts a more sophisticated analytical technique, and most importantly, demonstrates the feasibility of the NIRS for *in vivo* applications.

## Materials and Methods

On the medial femoral trochlear ridges of both femoropatellar joints of adult Shetland ponies (*N* = 7, 6 females and 1 male, Age = 8.8 ± 3.5 years), two cylindrical (*d* = 10 mm) chondral lesions were surgically created, resulting in a total of 28 lesions.^27^ Lesions were filled with four different experimental repair techniques (three varieties of gelatin methacryloyl (GelMA) hydrogels or fibrin glue) to investigate their potential—please refer to our earlier paper for further details on the repair techniques (open access).^27^ The positions of these four experimental repairs were randomized equally between the four predetermined anatomical locations (left proximal, left distal, right proximal, and right distal femoral trochlea) to account for any variability influenced by the anatomical location. The ponies were sacrificed after 12 months follow-up and the tissue surrounding the repair sites was evaluated using a conventional arthroscope and a novel arthroscopic NIRS probe. Subsequently, samples were extracted after removing the skin and overlying tissues of the joint and frozen (at − 20°C) until required for further analysis. No substantial changes in cartilage structure or composition are expected by subjecting the samples for a freeze–thaw cycle.^23^ In addition, samples from matching anatomical location were extracted from the joints of healthy adult Shetland ponies (*N*_*control*_ = 3, Age = 10.3 ± 4.7 years) to enable comparison between the two groups: experimental and control ponies. The Ethics Committee of Utrecht University for Animal Experiments in compliance with the Institutional Guidelines on the Use of Laboratory Animals approved the study (Permission DEC 2014.III.11.098). The measurements were carried out in a surgical theatre at the Department of Equine Sciences, Utrecht University, The Netherlands. The control ponies were acquired from a slaughterhouse in Nijkerk, The Netherlands and thus no gender information was available. Unlike in humans,^13^ there are thus far for the equine species no indications for gender-related differences in the susceptibility to orthopaedic diseases, such as OA, nor in differences related to the healing capacity of articular injuries.

**Figure 1:**
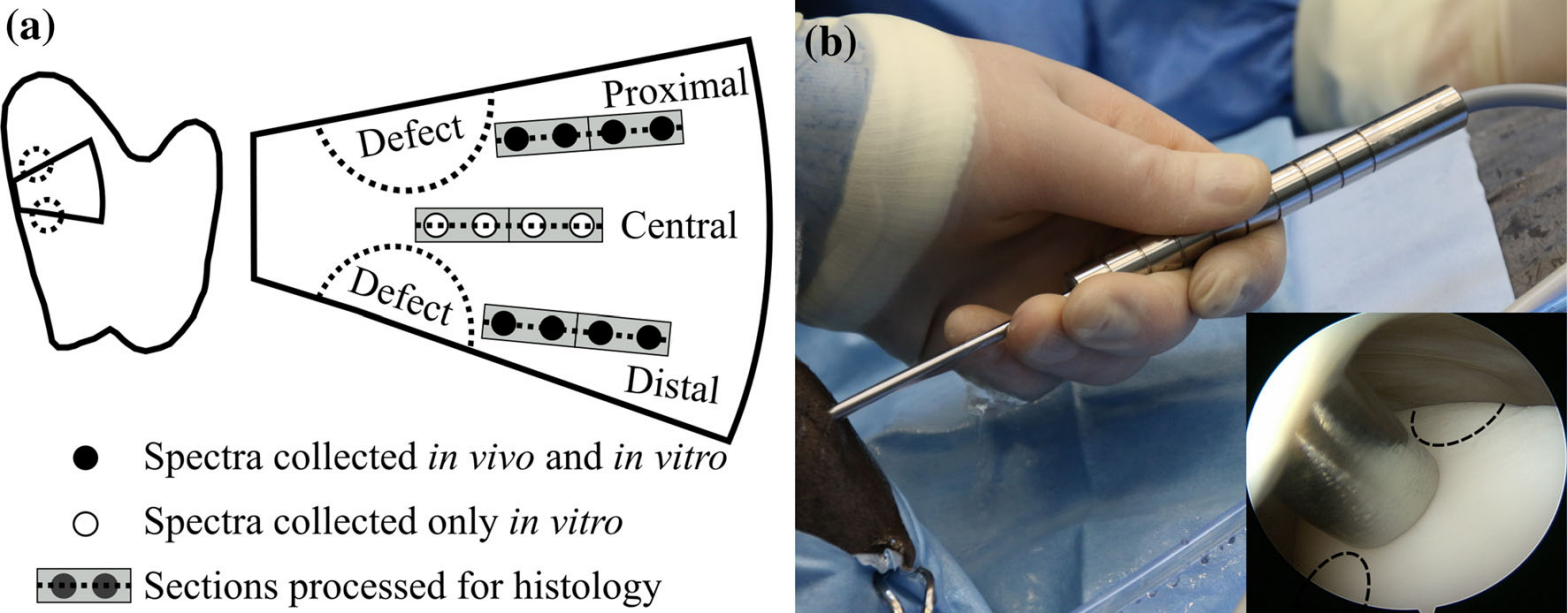
(a) The measurement locations (black and white circles) on an osteochondral sample along with the locations (grey rectangles) from which tissues were extracted for histological analysis (dashed line on a grey rectangle). On each measurement line (proximal, central, and distal), the measurement location closest to a defect was denoted as location 1 and the farthest as location 4. (b) The novel arthroscopic NIRS probe during *in vivo* spectral acquisition with the probe tip in contact with cartilage surface (inset, at Distal location 3). The distance between the locations of spectral acquisitions was equal to the outer diameter (*d *= 3.25 mm) of the NIRS probe.

Arthroscopic NIR spectra (*n *= 108) were acquired by an experienced board-certified equine surgeon (with experience of > 500 arthroscopies, Diplomate European College of Veterinary Surgeons) under the guidance of a traditional arthroscope (4 mm, 30° inclination, Synergy HD3, Arthrex, Naples, FL, USA). The NIRS system consisted of spectrometers (AvaSpec-ULS2048L, *λ* = 0.35–1.1 *µ*m, resolution = 0.6 nm and AvaSpec-NIR256-2.5-HSC, *λ* = 1.0–2.5 *µ*m, resolution = 6.4 nm, Avantes BV, Apeldoorn, The Netherlands), a light source (AvaLight-HAL-(S)-Mini, *λ* = 0.36–2.5 *µ*m, Avantes BV), and the custom-designed arthroscopic probe manufactured by Avantes BV. The reusable stainless-steel probe (*d *= 3.25 mm) is sterilisable in an autoclave at 121 °C with the probe tip window (*d* = 2 mm) containing 114 optical fibres (*d* = 100 *µ*m) with 14 fibres used to collect the reflected and scattered light. Eight locations surrounding the cartilage repair sites were measured (Fig. 1) with the NIRS probe held in perpendicular contact with the cartilage surface. At each measurement point, 15 spectra were recorded, each being the average of ten successive spectra; the total duration of data acquisition was 2.4 s per measurement location. In addition, the traditional arthroscope and palpation hook were utilized to evaluate the measurement locations according to the ICRS scoring system.^7^ Arthroscopic images were recorded during the measurements to enable reliable location tracking. For joint distension, Ringer’s solution (Fresenius, Bad Homburg v.d.H., Germany) containing sodium chloride (8.6 g/L), potassium chloride (0.3 g/L), and calcium chloride (0.33 g/L) was used.

The NIR spectra (*n *= 236; experimental and control groups) were re-acquired *in vitro* under similar conditions as during arthroscopy procedure, i.e., including NIRS hardware, immersion solution, and temperature, apart from using the conventional arthroscope for navigation. Three successive spectra, each being an average of 10 spectra, were acquired at each measurement location. The different acquisition protocols for *in vivo* and *in vitro* measurements were utilized to account for operator-based movement and hardware-related variation, respectively.

In addition to spectral measurement, cartilage thickness was determined by imaging with optical coherence tomography (OCT, *λ* = 1305 ± 55 nm, axial resolution < 20 *µ*m, lateral resolution 25–60 *µ*m; Ilumien PCI Optimization System, St. Jude Medical, St. Paul, MN, USA).^27^

### Histological Processing and Imaging

Histological sections were prepared for each NIRS measurement location (Fig. 1a) by the process of fixing samples in formalin, subsequent decalcification in EDTA, and embedding in paraffin. For each NIRS measurement location, three sections were prepared with a microtome for DD (thickness =3 *µ*m) and PLM (thickness =5 *µ*m) imaging.^26^ In the analysis, full-thickness and superficial (first 25%) cartilage PG content and collagen orientation angle were determined. The first 25% of cartilage was chosen to represent a layer in which collagen orientation changes from parallel orientation towards perpendicular orientation relative to cartilage surface.^29^ This layer represents the conventional superficial and middle zones that are traditionally determined based on profiles of collagen orientation and optical retardance; however, due to the disruption of the collagen network in some of the present samples, this was not always feasible.

**Figure 2:**
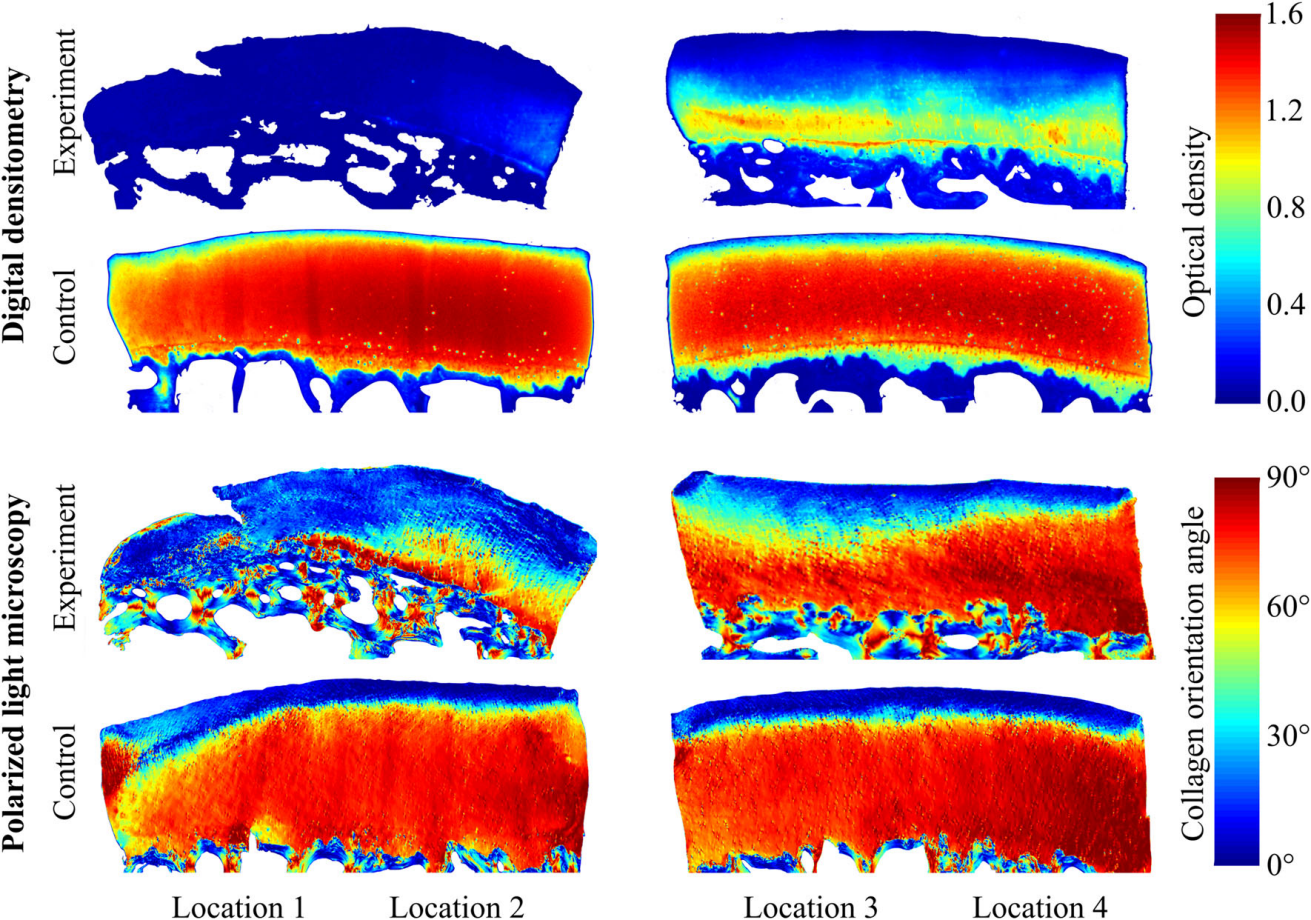
Example digital densitometry (optical density, i.e., proteoglycan content) and polarized light microscopy (collagen orientation angle) images of the experiment (worst case) and control (best case) groups from matching anatomical sites (distal locations 1-4). The arthroscopic photo shown as an inset in Fig. 1b was recorded during NIRS acquisition of the worst case at Distal location 3.

To determine cartilage PG content *via* optical density (OD), the sections were stained with safranin-O and imaged using a DD system (Fig. 2). The system consists of a light microscope (Nikon Microphot-FXA, Nikon Co., Tokyo, Japan), equipped with a monochromatic light source and a 12-bit CCD camera (ORCA-ER, Hamamatsu Photonics K.K., Hamamatsu, Japan). The system was calibrated with filters having OD ranging from 0 to 3. In the analysis, the average OD profile of each location was determined by extracting depth-wise profiles perpendicular to the cartilage surface, interpolating them to 500 points, and averaging the interpolated profiles.

An Abrio PLM system (CRi, Inc., Woburn, MA, USA), mounted on a conventional light microscope (Nikon Diaphot TMD, Nikon Inc., Shinagawa, Tokyo, Japan), was used to determine collagen orientation in the samples (Fig. 2). The system consists of a green bandpass filter, a circular polarizer, and a computer-controlled analyser, composed of two liquid crystal polarizers and a CCD camera. In the orientation images, 0° corresponds with an orientation parallel to the cartilage surface and 90° with a position perpendicular to the cartilage surface. Similarly to the DD analysis, the depth-wise profiles perpendicular to cartilage surface were determined, followed by interpolation and averaging.

### Spectral Pre-processing

Spectral measurements often include environmental or hardware-related noise; therefore, signal pre-processing is essential for ensuring reliable data for modelling. The acquired spectra were pre-processed with several techniques, including smoothing, 1st derivative, and 2nd derivative pre-processing. Due to using two detectors with varying resolution, the wavelength regions were pre-processed separately with a 3rd degree Savitzky-Golay^28^ algorithm using 139 points (0.08 *µ*m, spectrometer: AvaSpec-ULS2048L) and 41 points (0.26 *µ*m, spectrometer: AvaSpec-NIR256). These window lengths ensured sufficient noise reduction without any substantial loss of essential information.

### Outlier Detection

**Figure 3:**
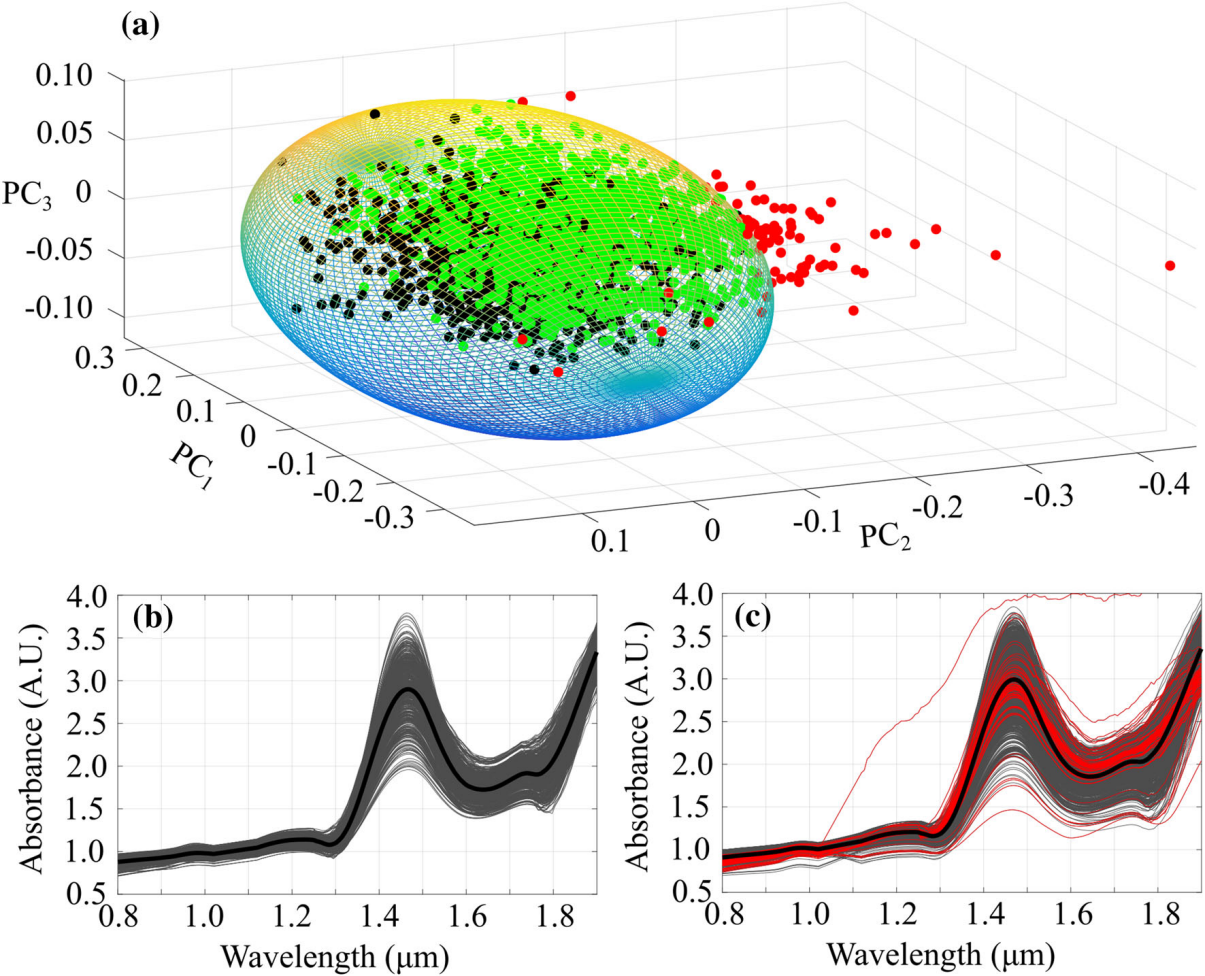
Minimum volume ellipsoid (MVE, a) calculated from the scores of *in vitro* (black) NIR spectral measurements. In addition, scores of *in vivo* spectral measurements within the MVE are presented (green) along with the outliers (red). In addition, smoothed *in vitro* (b) and *in vivo* (c) spectra with average spectra (black) are presented. In subfigure (c), outlier spectra are shown in red.

In our previous study,^27^ arthroscopic spectra were found to have contributions from the arthroscopic light source (0.40–0.75 *µ*m); therefore, a spectral region of 0.40–0.80 *µ*m was discarded from the analysis. To address the possibility of non-optimal contact between NIRS probe and cartilage surface during arthroscopic measurements, a minimum volume ellipsoid (MVE) approach was adopted. PCA was utilized to calculate the first three principal scores of pre-processed *in vitro* spectra, followed by calculation of scores for *in vivo* spectra by projecting them to the first space. As *in vitro* spectra (green, Figs. 3a and 3b) were always recorded with a reliable contact between the probe and tissue in a controlled environment, these scores were utilized to calculate the MVE (Fig. 3a). *In vivo* spectra falling outside the ellipsoid (red, Figs. 3a and 3c) were classified as outliers. In the analysis, the radii of MVE were uniformly altered to investigate their effect on NNs’ prediction performance.

### Data Preparation

To ensure well-generalized models, fivefold cross-validation was employed with 2 out of 10 ponies used as the validation set (each pony was used once in the validation). With each reference property, the ponies for the validation set were selected based on the pony-specific average value, i.e., in the first fold ponies with lowest and sixth lowest average values were assigned to the validation set (1–6, 2–7, *etc*.). This division was adopted to ensure representative calibration and validation sets (range of data), along with ensuring that the calibration set had no dependent information in the validation set. In addition, the reference properties and spectra were scaled with the MinMaxScaler and StandardScaler functions in the sklearn package,^21^ respectively.

### Network Design and Training

As NNs require substantial amount of data to ensure well-generalizable models, data augmentation was adapted in this study by including the three repeated *in vitro* spectra from each location in the modelling. In addition, several studies have highlighted the advantage of ensemble networks over a single network; thus, two separate network architectures were developed. To further optimize the modelling, the networks were initialized with three random seeds (7, 14, and 21). Therefore, the performance of the ensemble network was evaluated by averaging the outputs of these six variants. The network architectures were optimized by monitoring the overall performance of the validation sets.

The first network architecture was adapted from Bjerrum *et al.* by employing 1D-CNNs.^5^ The optimal network had two 1D convolutional layers (layer1_kernel_ = 8, layer1_filter_ = 64, layer2_kernel_ = 64, layer2_filter_ = 16) with ReLu activations.^18^ The outputs were flattened, followed by a dropout layer (ratio = 0.75), a fully connected dense layer (neurons = 128) with a ReLu activation, and the output layer with a linear activation. The second network architecture included a dense layer (neurons = 120) with a sigmoid activation, a dense layer (neurons = 60) with a ReLu activation, a dropout layer (ratio = 0.20), a dense layer (neurons = 15) with a ReLu activation, and the output layer with a linear activation. Root mean square errors (RMSE) of validation sets were used to evaluate the performance of both network architectures during the training. The training was performed using the Adam optimizer in Keras.^11^ To limit the chance of overfitting, Keras EarlyStopping and ReduceLROnPlateau callbacks were utilized to halt training if no improvement was achieved after 25 epochs and to reduce the learning rate after 20 epochs, respectively.

### Statistics

The performance of the ensemble network calibration (*in vitro*), validation (*in vitro*), and test (*in vivo*) sets was calculated by averaging the location-specific predictions, i.e., with *in vitro* data, the predictions of three repetitions were averaged and, with the *in vivo* data, the predictions from non-outlier spectra were averaged. Due to a non-normal distribution of reference parameters (Shapiro–Wilk test, *p *< 0.0001), non-parametric tests were used. SPSS (Version 25, SPSS Inc., IBM Company, Armonk, NY, USA) was used in the statistical analysis. Statistical significance of differences in tissue properties between experimental and control groups was investigated using the Mann–Whitney U test with *p* < 0.05 as the limit for statistical significance. For testing the statistical differences between the measured and predicted reference values, a Wilcoxon signed rank test was used with *p* < 0.05 as the limit for statistical significance. Linear Mixed Model analysis was performed to determine underlying causes for the variability of reference properties by using the anatomical location (distal, proximal), repair group (1–4) and control, and measurement location (location 1–4) as fixed effects, whereas pony and leg (pony × leg) (left or right) were utilized as random effects.

## Results

In the arthroscopic evaluation, the cartilage surrounding the repair sites was visually intact with no signs of cartilage fibrillation or lesions. However, during cartilage palpation, all measurement locations around the repair were soft in comparison to native cartilage. Furthermore, cartilage stiffness was observed to increase when moving away from the repair sites. Thus, the measurement locations were systematically denoted with the score of ICRS 1a.

**Figure 4:**
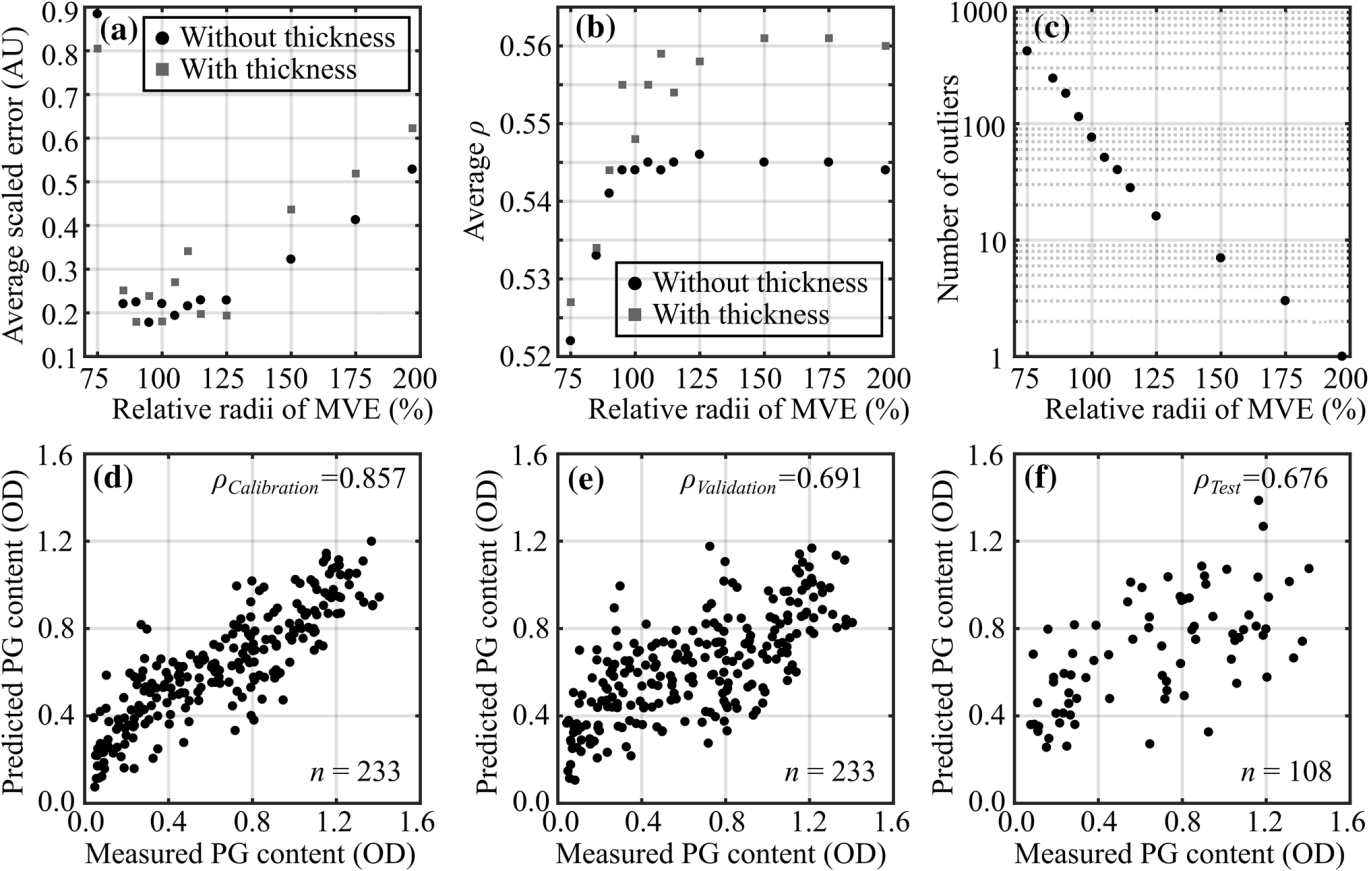
Detection of outlier *in vivo* spectra was performed by calculating the minimum volume ellipsoid (MVE) of the first three principal scores of *in vitro* spectra. For cartilage reference properties, the average scaled root mean square error (RMSE, a), average Spearman’s rank correlation (*ρ*, b), and the number of outliers (c) are presented as a function of relative MVE radii with and without cartilage thickness. In addition, the relationships between measured and predicted full-thickness proteoglycan (PG) content (optical density, OD) for calibration (*in vitro,* d), validation (*in vitro,* e), and test (*in vivo*, f) sets are presented.

During initial preliminary modelling, 1st derivative pre-processing exhibited systematically better performance over the other pre-processing methods and was, thus, selected as the optimal pre-processing method in the final analyses. In the outlier detection, the best performance (smallest average RMSE of test set) was observed with slightly larger MVE (radii of 105%, Figs. 4a and 4c) with 51 outliers (out of 1620 collected during the arthroscopy). The average error of test sets substantially increased (correlation decreased) after reduction of radii by 25% (100% → 75%), indicating extensive rejection of good spectra. A less substantial difference was observed when the radii were increased, thus indicating a low number of outlier spectra during the arthroscopic measurements.

**Figure 5:**
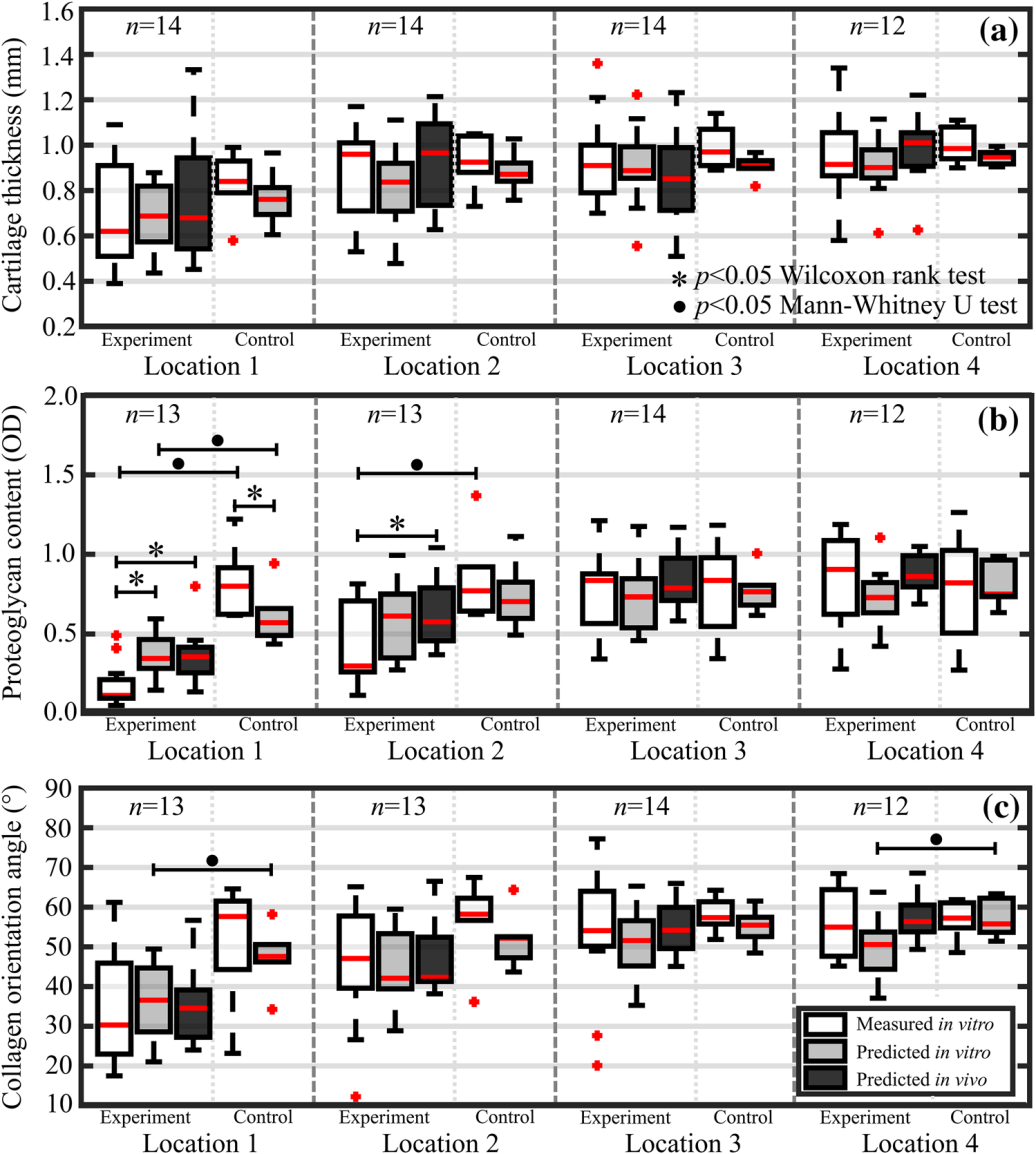
Boxplots for experimental and control groups with median (red line), quartiles (25% and 75%), and outliers (red crosses) of *in vitro* measured (white bars), *in vitro* predicted (grey bars), and *in vivo* predicted (black bars) for the 4 distal locations at increasing distances from the lesion. Values are presented for cartilage thickness (a), full-thickness proteoglycan content (b), and full-thickness collagen orientation angle (c). For each location, experiment and control groups had 12–14 and 6 measurements, respectively.

The performance of the trained ensemble networks with both validation (*in vitro*) and test (*in vivo*) sets for the cartilage reference properties, including cartilage thickness, full-thickness and superficial PG content and collagen orientation angle, is presented in Table 1. In addition, predictions based on validation and test sets enabled a comparison between the experimental and control groups (Fig. 5).

**Table 1:** Average cartilage reference properties and statistics on network performance, including Spearman’s rank correlation (*ρ*, *p < 0.001), root mean square error (RMSE), and normalized RMSE (NRMSE)

Parameter	Cartilage thickness (mm)	Full-thickness PG content (OD)	Superficial PG content (OD)	Full-thickness collagen orientation angle (°)	Superficial collagen orientation angle (°)
Mean (range)	0.82 (0.14–1.36)	0.66 (0.04–1.41)	0.37 (0.04–1.17)	46.9 (10.1–77.2)	17.1 (3.6–56.0)
**Calibration** (***in vitro***)					
*ρ*	0.888*	0.857*	0.800*	0.781*	0.582*
RMSE	0.108	0.208	0.174	10.967	7.994
NRMSE	8.8%	15.3%	15.4%	16.4%	15.2%
**Validation** (***in vitro***)					
*ρ*	0.797*	0.691*	0.650*	0.626*	0.327*
RMSE	0.141	0.274	0.220	13.111	9.495
NRMSE	11.5%	20.1%	19.5%	19.6%	18.1%
**Test** (***in vivo***)					
*ρ*	0.596*	0.676*	0.613*	0.574*	0.316*
RMSE	0.191	0.296	0.248	13.439	11.642
NRMSE	17.2%	21.8%	22.0%	20.0%	22.2%

**Figure 6:**
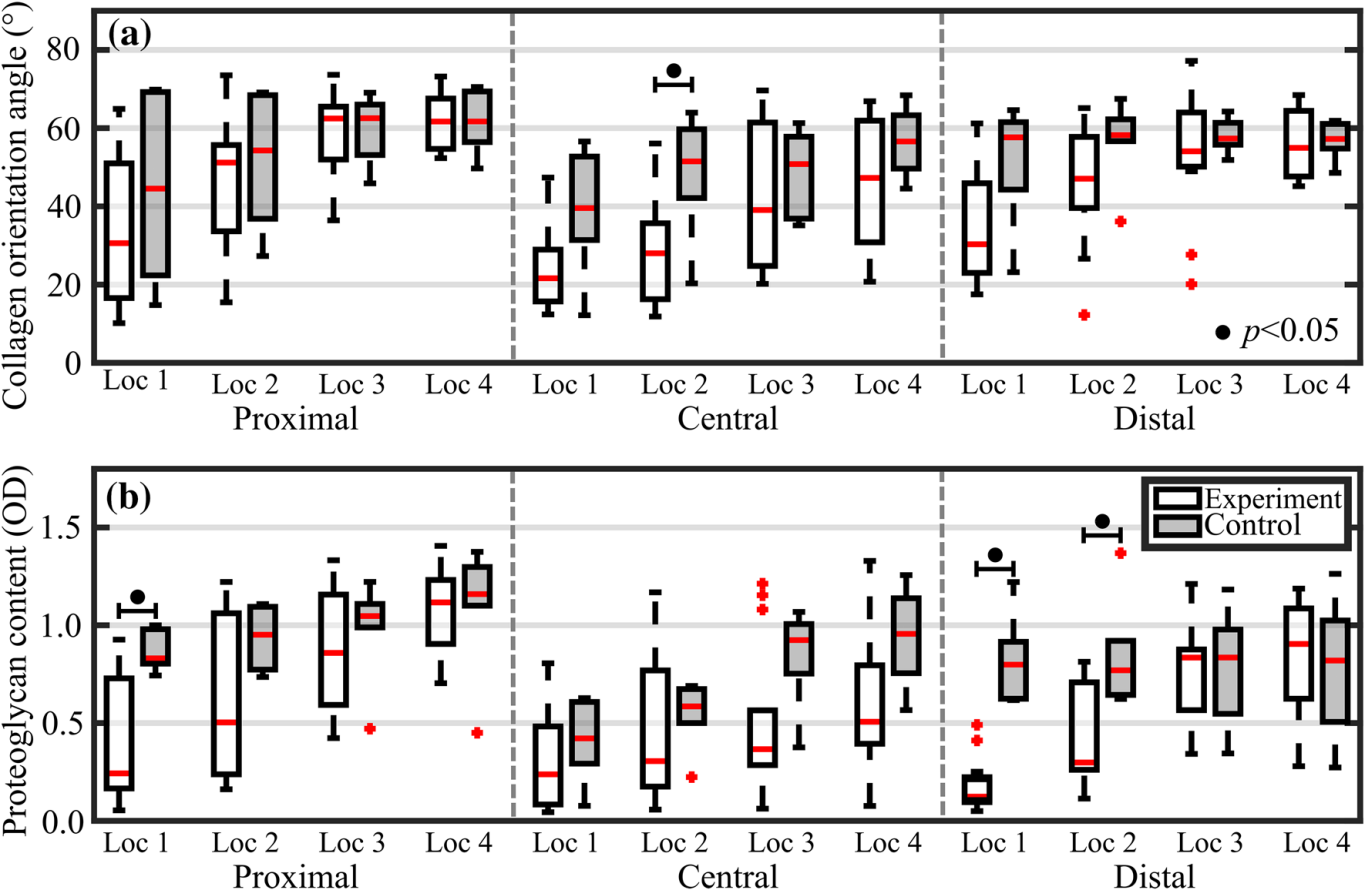
Boxplots for experimental and control groups with median (red line), quartiles (25 and 75%), and outliers (red crosses) of measured full-thickness collagen orientation angle (a) and proteoglycan content (b) for the proximal, central, and distal sites.

When comparing the measured reference values of experimental and control groups (Fig. 6), significant (*p *< 0.05) differences in full-thickness PG content were observed in locations closest to lesions on proximal and distal sites. However, no difference was apparent with full-thickness PG content or collagen orientation angle at locations further away from lesions, thus possibly indicating that locations closest to the lesion are more vulnerable to excessive stresses and strains and, hence, progressive degeneration as also demonstrated by Venäläinen *et al.*^38^ However, contributions from other factors, including inflammatory signalling and cell-mediated factors, could not be accounted for in the analysis. Based on the linear Mixed Model, the measurement location had by far the most significant effect (*p *< 1E−13 with all reference properties), whereas anatomical location (thickness: *p *= 0.316, PG: *p *= 5.2E−6, and collagen orientation angle: *p *= 0.442) and repair group (thickness: *p *= 0.026, PG: *p *= 0.040, and collagen orientation angle: *p *= 0.646) had significant effects in few occasions. In pairwise comparison between healthy (control group) and different repair groups (experimental group), a significant difference (*p *= 0.034) was observed only with cartilage PG content with one repair group (*GelMa reinforced*).

## Discussion

In this study, an arthroscopic NIRS was introduced, for the first time, for *in vivo* evaluation of cartilage thickness, PG content, and collagen orientation angle. Furthermore, no previous study has utilized 1D-CNNs to investigate the relationship between cartilage NIR spectra and reference properties; although, these relations have been previously demonstrated in several *in vitro* studies.^3^,^25^,^26^ The spectral region utilized in this study was similar or wider compared to the previous studies. The findings presented here demonstrate the clinical potential of NIRS for the evaluation of cartilage thickness, composition, and structure. Particularly, the prediction of superficial cartilage PG content could prove useful in detecting compromised cartilage prior to visible signs of degradation. The presented technique has the potential to improve the arthroscopic assessment of cartilage integrity. This could lead to better staging of OA and aid decision-making in cartilage repair surgery, for example, when selecting suitable autograft for mosaicplasty surgery or when assessing the boundaries of focal cartilage defects and healthy cartilage.

Significant differences in collagen orientation angle and PG content between the experimental and control groups were only observed at the locations closest to lesions (Fig. 6). Although there were no other significant differences between the two groups, the observable difference substantially decreased when moving away from the lesions, with no apparent difference between both groups at the farthest location (location 4). The application of NIRS could, therefore, enable demarcation of the boundary between healthy and compromised cartilage tissue when performing repair arthroscopies. However, prior to clinical application, a substantial cartilage reference library would need to be collected to establish the normal variance of different anatomical locations. Linear mixed model analysis revealed that the type of repair strategies had no substantial effect on cartilage properties relative to the effect of measurement location (i.e., distance from the repair). Cartilage tissue closest to the repair site has been exposed to higher than normal stresses and strains, potentially accelerating the tissue degeneration. This observation is supported by the findings of Venäläinen *et al.*^38^ Our findings are in line with those of Kaul *et al.*, who reported differences between repair tissue and adjacent articular cartilage after failed marrow-stimulation techniques (microfracture and Pridie drilling).^15^ In that study, the surrounding cartilage showed signs of early to severe OA after 8 months.

Spahn *et al.* and Hofmann *et al.* have previously applied NIRS in human arthroscopies^14^,^32^ and compared the reliability of the technique with scores from other modalities. Specifically, Spahn *et al.* reported spectral changes with varying severity of cartilage lesions^32^ and Hofmann *et al.* showed that NIRS values (based on a ratio of two spectral peaks^14^,^31^,^32^) correlate with KOOS (Knee Injury and Osteoarthritis Outcome Score^24^). The NIRS value varied between 0 (degenerated cartilage) and 100 (healthy cartilage) and was suggested to indicate diminished stiffness^17^,^33^ and altered water content.^31^,^33^ In addition to the suboptimal analysis technique, the studies utilized a narrower spectral region (1.1–1.7 *µ*m) compared to the present study (0.8–1.9 *µ*m). The inclusion of shorter and longer wavelengths within the wider spectral region enables evaluation of subchondral bone^27^ and more specific evaluation of superficial layer, respectively, due to the wavelength-dependent penetration of light into the tissue.^20^

Previous studies have reported the relationship between native cartilage PG content and its NIR spectra.^3^,^26^ Afara *et al.* demonstrated the potential of PG mapping with a similar spectral region (0.8–2.5 *µ*m) using bovine samples.^3^ Although the reported correlations were relatively higher, the study utilized PLSR with leave-one-out cross-validation, which is not as robust as *k*-fold cross-validation, since the calibration set always included measurements from the same joint as the validation (left out sample). In our previous* in vitro* study,^26^ combination of a narrower spectral region (0.7–1.1 *µ*m) and shallow NNs was demonstrated with samples from equine cartilage, resulting in similar correlations as presented here. With superficial cartilage PG content, the ensemble NN achieved a lower prediction error (RMSE) compared to the shallow NN in our previous study. The previous study also utilized OCT-based image classification to improve the prediction accuracy of the shallow NNs.^26^ However, in the present study, all cartilage samples were assessed as visually intact based on conventional arthroscopic evaluation and the OCT images, making image-based classification impractical.

The relevant spectral features harnessed by the NNs for predicting the target (reference) tissue properties are due to the depth-dependent interactions (absorption, scattering, *etc*.) of NIR light with the tissue. These interactions are related to chemical bonds of cartilage constituents, i.e., water, PG, and collagen. The NIR region is mostly affected by the first, second, and third overtone vibrations arising from stretching, bending, and combination bands. The most common bonds of cartilage constituents are OH, CH, NH, and CO. As cartilage matrix mainly consists of water (up to 80%),^29^ the absorption of OH stretching and bending vibrations is most pronounced in the cartilage spectrum. Several optical windows (e.g., 1.10–1.23 and 1.40–1.55 *µ*m), not affected by the masking effect of water, have shown good prediction performance. The relationship between collagen orientation angle and NIR spectra is expected to arise from the birefringence property of cartilage, with healthy and intact collagen network reflecting more light back to the NIRS probe. With cartilage thickness, the NIR spectra are affected by the path length of light and the strong interaction at the interface of cartilage and bone (due to difference in refractive indices).^4^,^40^ The superior penetration depth of NIRS, compared to MIRS, promotes its application for evaluation of whole cartilage tissue in clinical arthroscopies. In contrast to MIRS, NIRS is also capable of providing information on the underlying bone through articular cartilage in both equine and humans.^1^,^27^ These studies showed that the visible and short NIR spectral regions are optimal for estimating bone properties, such as subchondral plate thickness and volume fraction, due to their better penetration depth compared to longer wavelengths.^20^

Although analyses of cartilage NIR spectra have mostly relied on conventional multivariate approaches (e.g., PCA and PLRS), recent studies have explored the potential of shallow NNs^26^,^27^ due to their ability to model complex non-linear features.^12^,^19^ In this study, deep NNs with two different architectures were developed and tested with positive outcomes. With deep learning, a substantial amount of data is required to ensure reliable optimization and to limit the chance of overfitting. To account for this constraint, cross-validation, dropout layers, and early stopping were utilized. In cross-validation, the division was based on pony level (i.e., calibration and validation set did not include data from the same pony), therefore ensuring that the validation set had independent data.

Outlier detection is essential in diagnostics to ensure reliable estimation of tissue properties.^27^,^30^ In our earlier study utilizing the same NIR spectral data,^27^ 8 out of 15 spectra were eliminated based on contributions from the light source of the conventional arthroscope during measurements. However, this does not account for the relative orientation of the NIRS probe to the arthroscope as inclination of NIRS probe tip (non-optimal contact) towards arthroscopic light source will result to stronger contributions compared to an inclination to opposite direction; therefore, the MVE approach was adopted. In the study of Spahn *et al.,*^30^ no explicit reasoning for outlier classification was given. The MVE approach, based on the controlled *in vitro* measurements, enables fast and effective detection of outliers as observed with the prediction results.

During the arthroscopic NIRS measurements, the narrow cavity of the equine joint restricted probe mobility and, therefore, limited the alignment between the probe and cartilage surface. However, the number of outlier measurements was relatively low (3.1%), demonstrating the clinical feasibility of the technique. The main limitation of this study was the contribution of the arthroscopic light source, as this limits the use of data from the visible spectral region, which may be useful for predicting subchondral and trabecular bone properties.^27^ As an animal model, equines were well-suited for this study due to similar cartilage thickness with humans.^16^ In addition, for example, racehorses suffer from joint injuries that are often evaluated and treated arthroscopically, thus demonstrating the usefulness of the technique in a relevant veterinary field.

In conclusion, NIRS in combination with deep NNs can be utilized for predicting cartilage thickness, PG content, and collagen orientation angle during *in vivo* arthroscopy, enabling differentiation between the experimental and control groups. Therefore, adaptation of NIRS may prove useful clinically, e.g. when arthroscopically selecting suitable allograft tissue for mosaicplasty surgery or when assessing the boundaries of focal cartilage defects and healthy cartilage.
